# The Predictive Value of Circulating Gal‐3 for New Stroke Events in Paroxysmal Atrial Fibrillation Patients Despite Oral Anticoagulation Medications

**DOI:** 10.1002/clc.70084

**Published:** 2025-02-04

**Authors:** Yihan Wang, Qianran Luan, Ying Dong, Xiaoming Zhu

**Affiliations:** ^1^ School of the Third Clinical Medical College Capital Medical University Beijing People's Republic of China; ^2^ Heart Center & Beijing Key Laboratory of Hypertension, Beijing Chaoyang Hospital Capital Medical University Beijing People's Republic of China; ^3^ Department of Gerontology The Third People Hospital of Chengdu Sichuan People's Republic of China

**Keywords:** atrial fibrillation, Galectin‐3, predictive biomarker, stroke

## Abstract

**Background:**

CHA2DS2‐VASc is used to assess the risk of stroke in patients with atrial fibrillation (AF) and guide anticoagulant treatment decisions, but it has limitations in accurately predicting stroke risk in individual patients. The objective of this study is to conduct a cohort study by assessing preoperative levels of Gal‐3 in paroxysmal AF patients, aiming to observe its correlation with the subsequent incidence of stroke events.

**Method:**

This study enrolled 197 patients with nonvalvular paroxysmal AF. Blood samples were taken to test Gal‐3 levels. All patients were followed up for 4 years after admission.

**Results:**

Compared to the nonstroke cases, serum levels of Gal‐3 were markedly elevated in stroke cases (7.08 [IQR, 4.60–10.96] vs. 17.34 [IQR, 8.28–20.31], *p* < 0.001). Gal‐3 yields a superior AUC (0.748, with a 95%CI of 0.681–0.807) compared to other classical stroke indices, such as BNP, CHA2DS2‐Vas score, and TNI. Remarkably, the Gal‐3 index exhibited a superior predictive capacity, yielding a significant incremental predictive value that surpassed the conventional risk factors (CHA2DS2‐VASc score) for stroke events, as evidenced by an IDI of 16.4% (*p* < 0.001) and an NRI of 34.7% (*p* = 0.002).

**Conclusion:**

The presence of Gal‐3 is an independent risk factor for stroke in patients with AF. Elevated levels of Gal‐3 have the potential to serve as a valuable biomarker for identifying incident strokes in AF patients. Furthermore, incorporating the assessment of Gal‐3 levels into the conventional CHA2DS2‐VASc score could significantly enhance its predictive accuracy for stroke in AF patients.

## Graphical abstract

Gal‐3 was found to be associated with the incidence of newly developed stroke in patients with nonvalvular paroxysmal atrial fibrillation.

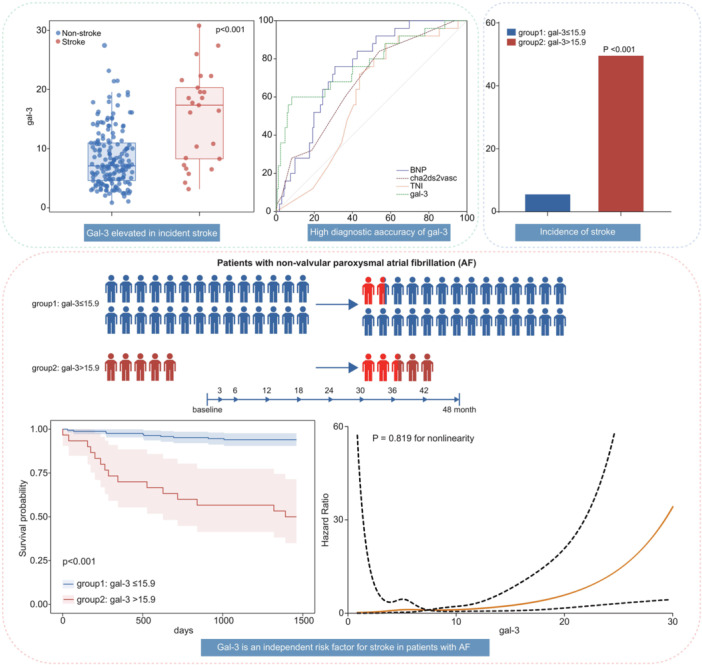

## 1 Introduction

Atrial fibrillation (AF) is the most common arrhythmia, and the overall lifetime risk is reported to range from 15% to 40% according to varied ethnicity [1]. Stroke and heart failure are the most significant adverse outcomes for patients with AF. Especially since the risk of stroke in AF patients is approximately three to five times higher compared to those without AF. Thanks to the advent and widespread use of oral anticoagulants (OACs), the incidence of ischemic stroke in patients with AF has significantly decreased [1]. Clinical risk score, such as CHA2DS2‐VASc, is utilized to estimate the risk of stroke in patients with AF and to guide decisions regarding anticoagulation therapy. The CHA2DS2‐VASc score, despite its widespread use in assessing stroke risk in AF patients, has several limitations, such as not fully reflecting all risk factors, ambiguity in the intermediate score range, persistent risk in low‐score patients, and variable predictive accuracy across different populations [2, 3]. In addition, although current stroke risk scores are practical, there are limitations regarding their capacity to accurately predict stroke risk in individual patients [3]. These limitations necessitate comprehensive clinical judgment and possibly additional tools for a more tailored preventive strategy [1]. Therefore, assessing the stasis in patients with AF using biomarkers related to thrombogenesis could provide a more accurate method to determine their stroke risk [2, 3].

Atrial cardiomyopathy, characterized by atrial remodeling, is increasingly considered not only to play a role in promoting tachycardia during AF but also in thrombogenesis [3]. Central to the hypothesis of atrial myopathy is the ongoing debate about whether thromboembolic stroke is triggered by arrhythmias or the condition of the atrial tissue itself. The ineffectiveness of rhythm‐control methods in lowering stroke risk, along with the absence of a consistent time link between paroxysmal AF and strokes during extended rhythm observation, suggests that fibrotic atrial cardiomyopathy could be playing a pivotal role in causing thromboembolism [4, 5]. While the specific pathways leading to stroke in AF are still not fully clear, indicators of atrial myopathy could significantly enhance the accuracy of current risk assessments for AF.

Galectin‐3 (Gal‐3) is a β‐galactoside‐binding lectin primarily produced by activated macrophages and belongs to the lectin family [6]. It has been previously related to atrial remodeling and proposed to have the ability to predict the onset, progression, and refractoriness of ablation [7, 8, 9]. Besides, Gal‐3 has previously been reported as a potential predictive biomarker for stroke [10, 11]. The underlying mechanism mainly focuses on its role in relating to atherosclerosis [12, 13, 14]. Additionally, reports have indicated that it may be significantly associated with forming left atrial (LA) appendage thrombi in patients with AF [15]. However, no studies currently show an association with cerebrovascular events following AF. Therefore, this study aims to conduct a cohort study by measuring preoperative levels of Gal‐3 in patients undergoing radiofrequency ablation for AF to observe its correlation with the subsequent incidence of stroke events. By using NRI/IDI analysis, the study will explore its ability to enhance the traditional CHA2DS2‐VASc score, further validating the hypothesis that the extent of cardiac remodeling is associated with the occurrence of stroke while also identifying a potential biomarker.

## 2 Methods

### 2.1 Study Subjects

The study included 197 consecutive nonvalvular paroxysmal AF patients admitted to the hospital. The age range was 19–75, with an average age of 68. All patients were diagnosed with AF through a 12‐lead electrocardiogram or a 24‐h holter monitor. Exclusion criteria included patients with cardiac valvular disease, recent acute coronary syndrome (within 6 months), severe hepatic or renal failure, chronic inflammation or cancer, recent cardiac surgery (within 30 days), and those with a life expectancy that precluded completion of the follow‐up. This study was approved by the Ethics Committee of Beijing Chaoyang Hospital (2024‐ke‐23), and all participants provided written informed consent. The Declaration of Helsinki conducted the study.

### 2.2 Laboratory and Clinical Information

Clinical baseline data were retrieved from the electronic medical records of the participants. All subjects underwent a trans‐thoracic echocardiogram within 3 days before their ablation procedures to assess LA size and left ventricular function. On the day of the ablation, blood samples were drawn to measure Gal‐3 level, high sensitive C‐reactive protein (hs‐CRP), B‐type natriuretic peptide (BNP) levels, and renal and liver function tests. Additional biochemical tests were conducted at our hospital's clinical laboratory center. Blood samples were collected in tubes containing ethylenediaminetetraacetic acid and were processed immediately or stored at 4°C if processing was postponed. All samples were handled within 24 h. To be specific, the plasma concentration of Gal‐3 was determined using enzyme‐linked immunosorbent assay (ELISA) kits (Immunoway (USA) KE1712), following the manufacturer's instructions.

### 2.3 Anticoagulation Strategy

Oral anticoagulation therapy was prescribed for patients with a CHA2DS2‐VASc score of 2 or higher. Oral anticoagulation therapy was initiated 3 weeks before the procedure for those undergoing catheter ablation. Postprocedure, anticoagulation therapy is typically continued for 3 months and then discontinued if there is no recurrence of AF for patients with a CHA2DS2‐VASc score of ≤ 1. For patients with a CHA2DS2‐VASc score ≥ 2 or those with recurrent episodes of AF, anticoagulation therapy is continued thereafter [16].

### 2.4 Follow Up

A total of 197 patients were followed up for 4 years after admission, with the primary outcome being the occurrence of stroke. Eight patients were lost to follow‐up: five due to personal reasons, two because contact was lost, and one due to death from a nonstroke‐related illness.

The time from the start of follow‐up to the occurrence of stroke was recorded for each patient. Patients had scheduled clinical visits and 12‐lead ECG at 3, 6, 12, and every 6 months after that. More frequent follow‐up visits were scheduled as needed for patients with any symptoms indicative of potential stroke. A new onset stroke is defined as detecting a novel lesion via computed tomography (CT) or magnetic resonance imaging (MRI) or the presence of clinical manifestations indicative of a stroke persisting beyond a 24‐h period [17]. Patients were divided into two groups during the follow‐up period: those with AF who experienced an ischemic stroke (*n* = 25) and those with AF but no stroke (*n* = 172).

### 2.5 Statistical Analysis

Data were summarized as mean ± standard deviation (SD), median (interquartile range), or number (percentages) where appropriate. When comparing the differences between the two groups, the Student's *t* test or Mann–Whitney U‐test was performed for continuous data, and the *χ* ^2^ test was conducted for categorical data. Spearman correlation analysis assessed the relationship between plasma Gal‐3 and BNP levels. Furthermore, receiver operating characteristics curve (ROC) analysis was carried out to determine the optimal cut‐off value of Gal‐3 for incident stroke with the maximum Youden index (sensitivity + specificity − 1). The areas under the ROC curve (AUCs) of BNP, CHA2DS2‐VASc score, and TNI were also assessed, and DeLong's test was performed to statistically compare AUCs.

Kaplan–Meier curves were plotted to describe the survival probability of stroke events by groups according to the optimal cut‐off of Gal‐3 and were compared using the log‐rank test. Cox proportional regression analysis was utilized to obtain the potential risk factors for incident stroke, and the results were recorded as hazard ratios (HRs) and 95% confidence intervals (95% CIs). Univariable cox regression analysis quantified the relationship between each predictor variable and the occurrence of stroke, selecting all clinical features that exhibited significant differences in Table 1. Subsequently, any variables that were found to be substantial (*p* < 0.05) in the univariable analysis were included in a multivariable cox regression analysis to determine the independent predictors of incident stroke. Meanwhile, restricted cubic spline (RCS) analysis with five knots (fifth, 25th, 50th, 75th, and 95th percentiles) was used to examine the nonlinear association between Gal‐3 and onset stroke. Nonlinearity was tested using the likelihood ratio test. Also, the net reclassification improvement (NRI) index and integrated discrimination index (IDI) index were calculated to estimate the incremental predictive value of Gal‐3 beyond the established risk factors of stroke (CHA2DS2‐VASc score). Statistical analyses were done with SAS V.9.4 (SAS Institute) and R version 4.3.1. *p* < 0.05 was considered statistical significance.

**Table 1:** Baseline characteristics of the study population.

	Total	Incident stroke	Nonstroke	*p* value
No.	197	25	172	
Age, years	66.9 ± 10.1	74.8 ± 7.9	65.8 ± 9.9	< 0.001
Male	111 (56.35%)	14 (56.00%)	97 (56.40%)	0.970
BMI, kg/m^2^	25.3 ± 3.8	24.7 ± 4.0	25.4 ± 3.8	0.343
cha2ds2vasc	3 (2–4)	4 (3–6)	3 (1–4)	0.003
Smoking	74 (37.56%)	11 (44.00%)	63 (36.63%)	0.477
Drinking	40 (20.30%)	6 (24.00%)	34 (19.77%)	0.623
Cerebral infarction	20 (10.15%)	6 (24.00%)	14 (8.14%)	0.029
Hypertension	136 (69.0%)	18 (72.00%)	118 (68.60%)	0.732
T2D	56 (28.43%)	7 (28.00%)	49 (28.49%)	0.960
Hyperlipemia	155 (78.68%)	23 (92.00%)	132 (76.74%)	0.082
RI	60 (30.46%)	12 (48.00%)	48 (27.91%)	0.041
PAD	10 (5.08%)	2 (8.00%)	8 (4.65%)	0.619
CAD	64 (32.49%)	11 (44.00%)	53 (30.81%)	0.188
MI	16 (8.12%)	2 (8.00%)	14 (8.14%)	0.981
Uric acid, umol/L	347.0 (301.0–418.0)	386.0 (341.0–461.0)	343(298.5–405.5)	0.044
TSH	1.77 (1.15–2.91)	2.93 (1.12–3.46)	1.77 (1.15–2.67)	0.204
hsCRP, mg/L	1.58 (0.80–3.38)	1.58 (0.57–4.77)	1.58 (0.81–3.36)	0.984
BNP, ng/L	222.0 (92.7–611.3)	710.7 (332.7–1607.0)	201.5 (82.2–442.6)	< 0.001
LAD, cm	39.0 (36.0–43.0)	41.0 (38.0–46.0)	39.0 (36.0–42.5)	0.079
Antiplatelet	43 (21.83%)	13 (52.00%)	30 (17.44%)	< 0.001
ACEI/ARB	85 (43.15%)	9 (36.00%)	76 (44.19%)	0.440
β‐blocker	96 (48.73%)	10 (40.00%)	86 (50.00%)	0.350
Statin	133 (67.51%)	20 (80.00%)	113 (65.70%)	0.154
Anticoagulation	192 (97.46%)	24 (96.00%)	168 (97.67%)	0.220
Ablation	153 (77.67%)	14 (56.00%)	139 (80.81%)	0.005

Abbreviations: BMI, body mass index; BNP, B‐type natriuretic peptide; CAD, coronary artery disease; hsCRP, high sensitivity C‐reactive protein; LAD, left atrial diameter; MI, myocardial infarction; No., number; PAD, peripheral artery disease; RI, renal insufficiency; T2D, type 2 diabetes mellitus; TSH, thyroid stimulating hormone.

## 3 Results

### 3.1 Serum Gal‐3 Level Was Significantly Elevated in Incident Stroke in Patients With Nonvalvular AF

In the present study, we consecutively enrolled 197 nonvalvular paroxysmal AF patients. Among them, 153 patients received radiofrequency ablation. Five out of 197 patients did not receive anticoagulation therapy throughout the follow‐up period, as their CHA2DS2‐VASc scores were < 2 (four patients had a score of 1, and one had a score of 0). Another 41 patients with a CHA2DS2‐VASc score of ≤ 1 (32 patients with a score of 1 and 10 with a score of 0) initially received anticoagulation therapy as they underwent radiofrequency ablation. We discontinued their oral anticoagulation 3 months after the ablation procedure, with exceptions to five patients who experienced recurrent AF episodes after the blanking period during follow‐up.

During a 4‐year follow‐up period, 25 patients out of a total cohort of 197 nonvalvular AF patients (mean [SE] age, 66.9 (10.1) years, 111 men [weighted 56.35%]) developed stroke events. Demographic, clinical, and biochemical data for the study population, stratified according to the presence of incident stroke, are shown in Table 1. Individuals with advanced age, a history of cerebral infarction, renal insufficiency, and receiving antiplatelet therapy were found to have a higher susceptibility to stroke. In addition, participants who experienced stroke consistently demonstrated notably higher CHA2DS2‐VAS scores, uric acid, and brain natriuretic peptide levels (*p *< 0.05 for all). Among the five patients who did not receive anticoagulation, one patient (with a CHA2DS2‐VASc score of 1) experienced a stroke (5% per year), while 24 out of 192 patients (3.1% per year) who received anticoagulation therapy, with an average CHA2DS2‐VASc score of 4, experienced a stroke.

**Figure 1:**
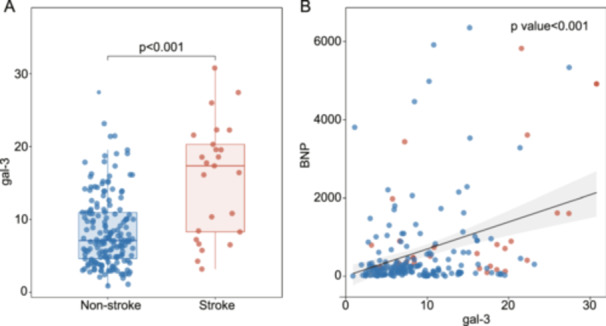
Analysis of plasma Gal‐3 levels in patients who progressed to stroke and those who did not progress to stroke. (A) Plasma levels of Gal‐3 were markedly elevated in stroked cases in comparison with the noncases; (B) Spearmen correlation analysis indicated a significant positive correlation between Gal‐3 concentrations and BNP levels (*r* = 0.27, *p* < 0.001). Gal‐3, Galectin‐3; BNP, B‐type natriuretic peptide.

Furthermore, compared to the nonstroke cases, serum levels of Gal‐3 were markedly elevated in stroke cases (7.08 [IQR, 4.60–10.96] vs. 17.34 [IQR, 8.28–20.31], *p* < 0.001, Figure 1A). Besides, a significant positive correlation was found between plasma Gal‐3 concentrations and BNP levels (Spearmen *r *= 0.27, *p *< 0.001, Figure 1B). In contrast, no definitive correlation was witnessed between Gal‐3 and the CHA2DS2‐VAS score (Spearmen *r* = 0.07, *p* = 0.35) or TNI (Spearmen *r* = −0.11, *p* = 0.130).

### 3.2 Gal‐3 Is an Independent Risk Factor for Incident Stroke in Patients With Nonvalvular AF

In addition, as depicted in Table 2, the univariate Cox regression analysis revealing that besides Gal‐3 (HR = 1.17, 95% CI: 1.11–1.23, *p* < 0.001), other traditional and well‐established risk factors such as age (HR = 1.13, 95% CI: 1.07–1.20, *p* < 0.001), CHA2DS2‐VASc score (HR = 1.39, 95% CI: 1.13–1.71, *p* = 0.002) and history of cerebral infarction history (HR = 3.37, 95% CI: 1.13–8.45, *p* = 0.010) were also significantly associated with the occurrence of stroke. In the multivariate Cox regression analysis model, after adjustment of confounding factors, three factors emerged as independent predictors of stroke incidence, including plasma Gal‐3 concentration (HR = 1.15, 95% CI: 1.07–1.25, *p* < 0.001), age (HR = 1.08, 95% CI 1.00–1.15, *p* = 0.041), and cerebral infarction history (HR = 5.54, 95% CI 1.25–24.53, *p* = 0.024), underscoring their pivotal roles in stroke risk assessment.

**Table 2:** Cox regression analysis.

Variables	Univariate		Multivariate	
	HR (95% CI)	*p* value	HR (95% CI)	*p* value
Gal‐3	1.17 (1.11–1.23)	< 0.001	1.15 (1.07–1.25)	< 0.001
Age	1.13 (1.07–1.20)	< 0.001	1.08 (1.00–1.15)	0.041
cha2ds2vasc	1.39 (1.13–1.71)	0.002	0.87 (0.59–1.28)	0.470
Cerebral infarction history	3.37 (1.35–8.45)	0.010	5.54 (1.25–24.53)	0.024
Hyperlipemia	3.31 (0.78–14.03)	0.105		
RI	2.23 (1.02–4.88)	0.046	1.87 (0.72–4.89)	0.200
Uric acid	1.04 (1.01–1.08)	0.019	1.05 (1.00–1.09)	0.057
BNP	1.01 (1.00–1.01)	0.031	1.00 (0.99–1.00)	0.286
LAD	1.08 (1.01–1.16)	0.025	1.06(0.99–1.13)	0.097
Antiplatelet	4.49 (2.05–9.84)	< 0.001	2.53(0.94–6.77)	0.065
Ablation	0.33 (0.15–0.72)	0.006	1.12 (0.41–3.10)	0.821

Abbreviations: BNP, B‐type natriuretic peptide; LAD, left atrial diameter; RI, renal insufficiency.

### 3.3 Gal‐3 Could be an Additional Biomarker for the Prediction of Incident Stroke in Patients With Nonvalvular AF

**Figure 2:**
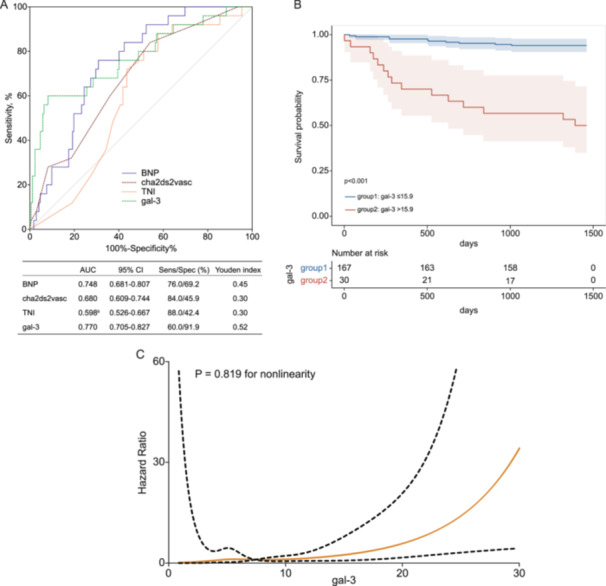
(A) AUC of Gal‐3, BNP, CHA2DS2‐VASc score, and TNI to identify subjects who progressed to stroke. Comparison of AUC in distinguishing stroke from nonstroke using Gal‐3, BNP, cha2ds2vasc score, and TNI, respectively. Sens, sensitivity; Spec, specificity; ROC curve, receiver operating characteristics curve; AUC, area under the ROC curve; Gal‐3, Galectin‐3; BNP, B‐type natriuretic peptide; TNI, cardiac troponins. ^b^: a significant difference was observed between Gal‐3 and TNI (*p* = 0.027) with DeLong's test. (B) Kaplan–Meier curve analysis. Kaplan–Meier curve analysis shows the freedom from stroke for patients with Gal‐3 ≤ 15.9 ng/mL and Gal‐3 > 15.9 ng/mL. The blue line represents ≤ 15.9 ng/mL, and the red line represents > 15.9 ng/mL. (C) Restricted cubic spline curve of the association between the Gal‐3 and incident stroke (*p* = 0.819 for nonlinearity).

Initially, the ROC curve of Gal‐3 for forecasting incident stroke is shown in Figure 2A. The serum Gal‐3 level demonstrates specific predictive utility for stroke onset with an optimal cut‐off value of 15.9, yielding a sensitivity of 60.0% and a specificity of 91.9% (maximum Youden's index). Furthermore, Gal‐3 yields a superior AUC (0.748, with a 95% CI of 0.681–0.807) compared to other classical stroke indices, such as BNP, cha2ds2vasc score, and TNI. Nevertheless, the statistical significance of this difference was only observed when Gal‐3 was compared to TNI, as evidenced by a *p *= 0.027 in DeLong's test.

Subsequently, the cohort is stratified into two groups: the low Gal‐3 group (Gal‐3 ≤ 15.9) and the high Gal‐3 group (Gal‐3 > 15.9) based on the optimal cut‐off value, and the Kaplan–Meier curve was produced for the two groups. In Figure 2B, the survival curves of the cohort with elevated Gal‐3 levels exhibited notably diminished outcomes compared to those with lower Gal‐3 levels, and differences between the two groups were verified by log‐rank test (*p *< 0.001). Further in‐depth analysis, utilizing the RCS model, demonstrated a linear correlation (*p *= 0.819 for nonlinearity, as illustrated in Figure 2C) between Gal‐3 levels and stroke incidences even after controlling for the covariates mentioned above (*p* < 0.05 in the univariate cox regression analysis). Remarkably, the Gal‐3 index exhibited a superior predictive capacity, yielding a significant incremental predictive value that surpassed the conventional risk factors (CHA2DS2‐VASc score) for stroke events, as evidenced by an IDI of 16.4% (*p* < 0.001) and an NRI of 34.7% (*p* = 0.002).

## 4 Discussion

This study investigated the relationship between serum Gal‐3 levels and the incidence of new strokes in patients with AF by measuring Gal‐3 and patient follow‐up. Our main findings include: (1) Gal‐3 is an independent risk factor for the occurrence of stroke in paroxysmal AF patients despite oral anticoagulation medications; (2) Adding the evaluation of elevated Gal‐3 levels to the traditional CHA2DS2‐VASc score can further enhance its predictive value for stroke. The results of this study not only suggest that Gal‐3 is a potential biomarker for stroke in AF patients, but also support the hypothesis that the degree of atrial remodeling may be a potential mechanism for stroke occurrence in these patients.

Studies have found that Gal‐3 levels are associated with different types of AF and displayed a gradient with higher levels in persistent and permanent AF compared to paroxysmal AF, with the highest levels observed in permanent AF [18]. Furthermore, elevated serum Gal‐3 levels in AF patients are also associated with an increased LA volume index, one of the key manifestations of atrial remodeling [19]. Additionally, studies have found a positive correlation between Gal‐3 and NT‐proBNP levels, a classical biomarker for atrial remodeling [20]. These findings collectively validate the association between Gal‐3 and atrial remodeling. Mechanistically, Gal‐3 may promote atrial fibrosis by enhancing extracellular matrix and cell adhesion, thus activating myofibroblasts and endocardial cells, which affects the fibrosis process and interferes with the progression of atrial remodeling [21]. The main finding of this study is that Gal‐3 can predict new‐onset strokes in AF patients, suggesting that besides its role in promoting atherosclerosis, as indicated by previous studies, it likely reflects the extent of atrial remodeling. Research has found that approximately one‐third of patients with both stroke and AF may not exhibit any clear episodes of AF in the months before their stroke. This indicates that although atrial arrhythmias can facilitate thrombus formation, there are many other factors involved in the relationship between stroke and AF [22]. Our findings further support this notion that atrial remodeling, rather than just irregular heartbeats, plays a significant role in the incidence of strokes in AF patients [3].

Ischemic stroke, a common complication of AF, often has a poor prognosis; assessing thromboembolic risk is critical in the appropriate management of patients with AF. Clinically, the CHA2DS2‐VASc score is primarily used to identify truly low‐risk AF patients who do not require anticoagulant therapy, thereby avoiding over‐treatment. However, its accuracy is limited and does not precisely reflect the extent of cardiac remodeling, making it less precise in predicting strokes following AF [23]. There is an increasing amount of research indicating that, in addition to clinical risk factors, serum biomarkers could be valuable in assessing stroke risk among patients with AF. BNP and TNI have emerged as the most promising among the biomarkers studied [3]. This study observed that serum levels of Gal‐3 are relatively higher in patients who experience a stroke following AF, indicating that Gal‐3 has a specific predictive value for the risk of ischemic stroke. In the present study, both the serum Gal‐3 and BNP levels showed significant predictive value for stroke incidences after AF by the ROC analysis. Interestingly, the BNP level significantly correlated with serum Gal‐3 levels. Although its standalone predictive utility is not as robust as that of the CHA2DS2‐VASc score, combining Gal‐3 with the CHA2DS2‐VASc score enhances the accuracy of predicting the risk of ischemic strokes in patients with nonvalvular AF.

### 4.1 Limitation

This study suffered several limitations. First, serum Gal‐3 levels might be influenced by other profibrotic and proinflammation conditions. However, in this study, we used strict selection criteria to exclude conditions that may influence the serum Gal‐3 levels, especially inflammation conditions like autoimmune diseases and acute phase of infectious or cardiovascular diseases. Second, we did not obtain direct evidence of cardiac thrombus formation to prove a cardioembolic stroke. However, as we have put in the introduction section, the potential mechanism for Gal‐3 to cause stroke was complex, incorporating both prothrombus and proatherosclerosis mechanisms. Besides, in the present study, we focus more on its clinical value than on exact molecular mechanisms. Nevertheless, further studies may be needed to link the prothrombus effect of Gal‐3 to cardioembolic strokes. Second, the present study reported a relatively higher rate of stroke incidence compared to previously reported rates. Part of the reason may be due to potential factors such as poor drug adherence and insufficient drug dosage, which are difficult to monitor and fully exclude others. Thus, caution should be taken when applying the results to a border population. Besides, Gal‐3 has been shown to be elevated in various conditions, including heart failure, chronic kidney disease, and inflammatory disorders, which may confound its use as a specific biomarker for stroke risk in AF patients. We have not included those patients in the present population. However, this raises the concern that Gal‐3, if used in isolation, may not be specific enough to solely predict stroke risk in all patients. Further studies are needed to understand better the factors that influence Gal‐3 levels and its role in the context of AF and stroke prevention.

## 5 Conclusion

Gal‐3 is an independent risk factor for stroke in AF patients despite oral anticoagulation medication. Elevated Gal‐3 levels could serve as a potential biomarker for incident stroke in AF patients. In addition, adding the evaluation of Gal‐3 levels to the traditional CHA2DS2‐VASc score could further enhance its predictive value for stroke in AF patients.

## Conflicts of Interest

The authors declare no conflicts of interest.

## Data Availability

The authors have nothing to report.

## References

[1] J. A. Joglar , M. K. Chung , A. L. Armbruster , et al., “2023 ACC/AHA/ACCP/HRS Guideline for the Diagnosis and Management of Atrial Fibrillation,” Journal of the American College of Cardiology 83 (2024): 109–279, 10.1016/j.jacc.2023.08.017. 38043043 PMC11104284

[2] J. J. Goldberger , R. Arora , D. Green , et al., “Evaluating the Atrial Myopathy Underlying Atrial Fibrillation: Identifying the Arrhythmogenic and Thrombogenic Substrate,” Circulation 132 (2015): 278–291, 10.1161/circulationaha.115.016795. 26216085 PMC4520257

[3] B. W. Calenda , V. Fuster , J. L. Halperin , and C. B. Granger , “Stroke Risk Assessment in Atrial Fibrillation: Risk Factors and Markers of Atrial Myopathy,” Nature Reviews Cardiology 13 (2016): 549–559, 10.1038/nrcardio.2016.106. 27383079

[4] D. J. Seiffge , V. Cancelloni , L. Räber , et al., “Secondary Stroke Prevention in People With Atrial Fibrillation: Treatments and Trials,” Lancet Neurology 23 (2024): 404–417, 10.1016/s1474-4422(24)00037-1. 38508836

[5] S. M. Al‐Khatib , N. M. Allen LaPointe , R. Chatterjee , et al., “Rate‐ and Rhythm‐Control Therapies in Patients With Atrial Fibrillation: A Systematic Review,” Annals of Internal Medicine 160 (2014): 760–773, 10.7326/m13-1467. 24887617

[6] V. Blanda , U. M. Bracale , M. D. Di Taranto , and G. Fortunato , “Galectin‐3 in Cardiovascular Diseases,” International Journal of Molecular Sciences 21 (2020): 9232, 10.3390/ijms21239232. 33287402 PMC7731136

[7] Q. Wang , L. Xu , Y. Dong , et al., “Plasma Galectin‐3 Is Associated With Progression From Paroxysmal to Persistent Atrial Fibrillation,” BMC Cardiovascular Disorders 21 (2021): 226, 10.1186/s12872-021-02043-0. 33934700 PMC8091760

[8] Q. Wang , W. Huai , X. Ye , et al., “Circulating Plasma Galectin‐3 Predicts New‐Onset Atrial Fibrillation in Patients After Acute Myocardial Infarction During Hospitalization,” BMC Cardiovascular Disorders 22 (2022): 392, 10.1186/s12872-022-02827-y. 36057558 PMC9440583

[9] Y. Takemoto , R. J. Ramirez , M. Yokokawa , et al., “Galectin‐3 Regulates Atrial Fibrillation Remodeling and Predicts Catheter Ablation Outcomes,” JACC: Basic to Translational Science 1 (2016): 143–154, 10.1016/j.jacbts.2016.03.003. 27525318 PMC4979747

[10] Z. Q. Cao , X. Yu , and P. Leng , “Research Progress on the Role of gal‐3 in Cardio/Cerebrovascular Diseases,” Biomedicine & Pharmacotherapy 133 (2021): 111066, 10.1016/j.biopha.2020.111066. 33378967

[11] A. Sayed , M. Munir , M. S. Attia , et al., “Galectin‐3: A Novel Marker for the Prediction of Stroke Incidence and Clinical Prognosis,” Mediators of Inflammation 2022 (2022): 2924773, 10.1155/2022/2924773. 35281427 PMC8904909

[12] A. C. MacKinnon , X. Liu , P. W. Hadoke , M. R. Miller , D. E. Newby , and T. Sethi , “Inhibition of Galectin‐3 Reduces Atherosclerosis in Apolipoprotein E‐Deficient Mice,” Glycobiology 23 (2013): 654–663, 10.1093/glycob/cwt006. 23426722 PMC3641797

[13] A. Edsfeldt , E. Bengtsson , G. Asciutto , et al., “High Plasma Levels of Galectin‐3 Are Associated With Increased Risk for Stroke After Carotid Endarterectomy,” Cerebrovascular Diseases 41 (2016): 199–203, 10.1159/000443022. 26812165

[14] Z. Gao , Z. Liu , R. Wang , Y. Zheng , H. Li , and L. Yang , “Galectin‐3 Is a Potential Mediator for Atherosclerosis,” Journal of Immunology Research 2020 (2020): 5284728, 10.1155/2020/5284728. 32149158 PMC7042544

[15] Z. Tang , L. Zeng , Y. Lin , et al., “Circulating Galectin‐3 Is Associated With Left Atrial Appendage Remodelling and Thrombus Formation in Patients With Atrial Fibrillation,” Heart, Lung and Circulation 28 (2019): 923–931, 10.1016/j.hlc.2018.05.094. 29861319

[16] J. A. Joglar , M. K. Chung , A. L. Armbruster , et al., “2023 ACC/AHA/ACCP/HRS Guideline for the Diagnosis and Management of Atrial Fibrillation: A Report of the American College of Cardiology/American Heart Association Joint Committee on Clinical Practice Guidelines,” Circulation 149 (2024): e1–e156, 10.1161/cir.0000000000001193. 38033089 PMC11095842

[17] M. R. Di Tullio , M. Qian , J. L. P. Thompson , et al., “Left Atrial Volume and Cardiovascular Outcomes in Systolic Heart Failure: Effect of Antithrombotic Treatment,” ESC Heart Failure 5 (2018): 800–808, 10.1002/ehf2.12331. 30015405 PMC6165930

[18] A. J. Camm , E. Simantirakis , A. Goette , et al., “Atrial High‐Rate Episodes and Stroke Prevention,” EP Europace 19 (2017): 169–179, 10.1093/europace/euw279. PMC5400077 28172715

[19] O. Goktekin , “Novel Fibro‐Inflammation Markers in Assessing Left Atrial Remodeling in Non‐Valvular Atrial Fibrillation,” Medical Science Monitor 20 (2014): 463–470, 10.12659/MSM.890635. 24651058 PMC3965288

[20] D. Hernández‐Romero , J. A. Vílchez , Á. Lahoz , et al., “Galectin‐3 as a Marker of Interstitial Atrial Remodelling Involved in Atrial Fibrillation,” Scientific Reports 7 (2017): 40378, 10.1038/srep40378. 28079145 PMC5228133

[21] P. Dilaveris , C. K. Antoniou , P. Manolakou , E. Tsiamis , K. Gatzoulis , and D. Tousoulis , “Biomarkers Associated With Atrial Fibrosis and Remodeling,” Current Medicinal Chemistry 26 (2019): 780–802, 10.2174/0929867324666170918122502. 28925871

[22] H. Kamel , P. M. Okin , M. S. V. Elkind , and C. Iadecola , “Atrial Fibrillation and Mechanisms of Stroke,” Stroke 47 (2016): 895–900, 10.1161/STROKEAHA.115.012004. 26786114 PMC4766055

[23] W. G. Zhu , Q.‐M. Xiong , and K. Hong , “Meta‐Analysis of CHADS2 Versus CHA2DS2‐VASc for Predicting Stroke and Thromboembolism in Atrial Fibrillation Patients Independent of Anticoagulation,” Texas Heart Institute Journal 42 (2015): 6–15, 10.14503/thij-14-4353. 25873792 PMC4378047

